# Identification of target genes of transcription factor activator protein 2 gamma in breast cancer cells

**DOI:** 10.1186/1471-2407-9-279

**Published:** 2009-08-11

**Authors:** He Ailan, Xiao Xiangwen, Ren Daolong, Gan Lu, Ding Xiaofeng, Qiao Xi, Hu Xingwang, Liu Rushi, Zhang Jian, Xiang Shuanglin

**Affiliations:** 1 Key Laboratory of Protein Chemistry and Developmental Biology of State Education Ministry of China, PR China; 2 College of Life Science, Hunan Normal University, Changsha, Hunan 410081, PR China

## Abstract

**Background:**

Activator protein 2 gamma (AP-2γ) is a member of the transcription factor activator protein-2 (AP-2) family, which is developmentally regulated and plays a role in human neoplasia. AP-2γ has been found to be overexpressed in most breast cancers, and have a dual role to inhibit tumor initiation and promote tumor progression afterwards during mammary tumorigensis.

**Methods:**

To identify the gene targets that mediate its effects, we performed chromatin immunoprecipitation (ChIP) to isolate AP-2γ binding sites on genomic DNA from human breast cancer cell line MDA-MB-453.

**Results:**

20 novel DNA fragments proximal to potential AP-2γ targets were obtained. They are categorized into functional groups of carcinogenesis, metabolism and others. A combination of sequence analysis, reporter gene assays, quantitative real-time PCR, electrophoretic gel mobility shift assays and immunoblot analysis further confirmed the four AP-2γ target genes in carcinogenesis group: ErbB2, CDH2, HPSE and IGSF11. Our results were consistent with the previous reports that ErbB2 was the target gene of AP-2γ. Decreased expression and overexpression of AP-2γ in human breast cancer cells significantly altered the expression of these four genes, indicating that AP-2γ directly regulates them.

**Conclusion:**

This suggested that AP-2γ can coordinate the expression of a network of genes, involving in carcinogenesis, especially in breast cancer. They could serve as therapeutic targets against breast cancers in the future.

## Background

The transcription factor activator protein-2 (AP-2) family consists of AP-2α, AP-2β, AP-2γ, AP-2δ and AP-2ε; they are retinoic acid inducible and developmentally regulated [1-6]. The AP-2 protein forms a unique modular structure containing an N-terminal proline- and glutamine-rich transactivational domain and a complex C-terminal helix-span-helix motif necessary and sufficient for dimerization and site-specific DNA binding. Through binding to a GC-rich recognition sequence in the regulatory regions of many genes, AP-2 protein provides a fundamental mechanism for regulating gene expression and involving into lots of cellular processes [7,8]. AP-2 proteins have been demonstrated to participate in the regulation of various signaling pathways during development, cell growth, differentiation and apoptosis by mouse knock-out studies [9-15]. AP-2 proteins are also essential for maintaining cellular homeostasis, and play a strict regulation role in the battle between normal cell growth and neoplasia [16,17].

AP-2 proteins have an essential role in the regulation of normal breast development [18], and are also involved in carcinogenesis, especially in breast cancer [19]. AP-2γ has been shown to participate in the regulation of ErbB2 [1] and estrogen receptor (ER) α [20], both of which contain binding sequence of AP-2γ in their promoter region and can promote tumorigenesis and metastasis [21]. On the other hand, AP-2γ also induces p21 expression, arrests cell cycle, and inhibits the tumor growth of human breast carcinoma cells [22,23]. These data suggest that there is a dual role for AP-2γ in different mammary tumorigenic stages: it initially delayed the appearance of tumors, however then accelerated tumor progression [23]. Taken together, these findings provide evidence that AP-2γ participates in a complex biologic dynamics, including cell cycle progression, apoptosis, and tumor formation.

To further understand the complicated roles of AP-2γ played in development and carcinogenesis, it is necessary to search more downstream target genes regulated by AP-2γ, and find out how they are regulated by AP-2γ. In this study, we isolated the binding sites of AP-2γ on the whole genomic DNA from human breast cancer cell line MDA-MB-453 using chromatin immunoprecipitation (ChIP) method. ChIP technique can identify the binding sites of native transcription factors in a given cellular context and reveal potential target genes. We found 20 novel binding sites of AP-2γ in the genomic DNA of MDA-MB-453 cells. These sites are associated with genes involved in the regulation of carcinogenesis, metabolism or others. A combination of sequence analysis, reporter gene assays, electrophoretic gel mobility shift assays, quantitative real-time PCR (Q-RT-PCR), and immunoblot analysis validated and further defined the binding sites on four target genes (ErbB2, CDH2, IGSF11 and HPSE) in carcinogenesis group. The expressions of these four genes were confirmed to be significantly altered by silencing or overexpression of AP-2γ. Moreover, Q-RT-PCR analysis revealed the effect of transcriptional enhancing or suppression by AP-2γ for different genes. The novel target genes of AP-2γ might be used as therapeutic targets against breast cancer in the future.

## Methods

### Cell culture

Human breast cancer cells MDA-MB-453, MDA-MB-231 and HBL-100 were cultured under conditions of 37°C and 5% CO_2 _in RPMI 1640 (Gibco, Rockville, MD, Carlsbad, USA) supplemented with 10% fetal bovine serum, 100 U/ml penicillin, 100 μg/ml streptomycin and 5 μg/ml insulin.

### Chromatin immunoprecipitation (ChIP) assay

ChIP assay was performed using the EZ ChIP™ Kit (Catalog # 17–371, Upstate Biotechnology, Boston, USA) according to the manufacturer's instruction. Briefly, cells were grown on 100 mm plates to 85% confluence; formaldehyde was added to a final concentration of 1%, and the plates were incubated for 10 min at 37°C; the cross-linking reaction was stopped by the addition of 100 mM glycine containing protease inhibitors (Boehringer cocktail, Ingelheim, Germany). Cells were then collected by centrifugation and lysed in SDS lysis buffer (1% SDS, 10 mM EDTA, 50 mM pH 8.0 Tris-HCl, and protease inhibitors); after sonication, DNA was sheared into 0.3–2 kb fragments; insoluble materials were removed by centrifugation. The extracts were diluted 10-fold in ChIP dilution buffer (0.01% SDS, 1.1% Triton X-100, 1.2 mM EDTA, 16.7 mM pH 8.1 Tris-HCl, and 167 mM NaCl) and precleared by incubation with Salmon Sperm DNA-Protein A coated agarose beads to reduce nonspecific interactions. The precleared supernatants were incubated with 2 μg of rabbit polyclonal anti-AP-2γ antibody (sc-8977, Santa Cruz Biotech, Delaware, USA), or 2 μg of normal rabbit IgG (Catalog # 12-371B, Upstate Biotechnology) overnight at 4°C; immune complexes were collected using Salmon Sperm DNA-Protein A coated agarose beads. The immunoprecipitated complexes were washed once in low salt wash buffer (0.1% SDS, 1% Triton X-100, 2 mM EDTA, 20 mM pH 8.1 Tris-HCl, and 150 mM NaCl), once in high salt wash buffer (0.1% SDS, 1% Triton X-100, 2 mM EDTA, 20 mM pH 8.1 Tris-HCl, and 500 mM NaCl), once in LiCl wash buffer (0.25 M LiCl, 1% IGEPAL-CA630, 1% Sodium deoxycholate, 1 mM EDTA, and 10 mM pH 8.1 Tris-HCl) and twice in TE buffer (10 mM Tris-HCl, 1 mM EDTA, pH 8.0). Following extensive washing, the cross-linked DNA-protein complexes were eluted using 1% SDS, 0.1 M NaHCO_3 _and dissociated by incubation at 65°C for 4–5 hours. After treatment with RNase A (Roche Applied Science, Basel, Switzerland) and proteinase K (Roche Applied Science), DNA was purified using the Qiagen MinElute kit; the purified DNA fragments can be used directly for PCR amplification of the genomic fragments of interest, or cloned as pUC19-ChIP-DNA for sequencing and further experiments.

### AP-2γ-ChIP-fragment luciferase vectors

AP-2γ-ChIP fragments were excised from pUC19-ChIP-DNA plasmids and inserted into pTAL-Luc vector (Clontech, Mountain view, USA) using *Kpn*I and *Xho*I sites. Site-directed mutagenesis was performed on the GCCN_3 _GGC motif within the putative AP-2γ binding sites of R1-15, R3-36 and R4-28 fragments. Primers (Table 1) corresponding to both strands with mutated bases in the middle were designed to produce mutant R1-15, R3-36 and R4-28 by overlapping extension PCR [24]. Briefly, in the first PCR, two parallel reactions were performed using pTAL-ChIP-DNA vectors as template: one with vector sense primer upstream of insert and mutated insert antisense primer, the other with mutated insert sense primer (complementary to the mutated insert antisense primer) and vector antisense primer downstream of insert; the equal molar mixture of these two PCR products was taken as template for the second PCR using the two vector primers. Final PCR product was subcloned into upstream of the firefly luciferase gene at KpnI/XhoI sites of pTAL-Luc vector. All constructs were sequencing confirmed, and we numbered the position of the AP-2γ binding site in the AP-2γ-ChIP fragments.

**Table 1:** Oligonucleotides used for site-directed mutagenesis and electrophoretic mobility shift assay (EMSA)

Name	Sequence 5'-3'	Name pTAL-Luc vector
WtR3-36/HPSE(F)	GGTACCGGCCTGGACTGCAGACCT	
WtR3-36/HPSE(R)	CTCGAGGCATGCCTGCAGGTCAAA	pTAL-R3-36/HPSE
WtR4-28/CDH2(F)	GGTACCCTGCGATGGGGGAGAACA	
WtR4-28/CDH2(R)	CTCGAGTCCCCCCACGAGAAAAAA	pTAL-R4-28/CDH2
WtR1-15/IGSF11(F)	GGTACCTTCTGTCATTTTGGGTCT	
WtR1-15/IGSF11(R)	CTCGAGTGGTTTTATCTGTGTAAT	pTAL-R1-15/IGSF11
WtR3-1/ErbB2 (F)	GGTACCGTAAAACGACGGCCAG	
WtR3-1/ErbB2 (R)	CTCGAGAGGAAACAGCTATGAC	pTAL-R3-1/ErbB2
⋆WtR3-36-648/HPSE(F)	AAAAGTGCCCAGAGCCCATGA	
⋆MtR3-36-648/HPSE(F)	AAAAGTattCAGgGCCCATGA	pTAL-R3-36-648/HPSE
⋆WtR3-36-676/HPSE(F)	AATGTGGCCCTGGTCACTTAG	
⋆MtR3-36-676/HPSE(F)	AATGTGattCTGaTCACTTAG	pTAL-R3-36-676/HPSE
⋆WtR4-28-854/CDH2(F)	ATGCGACCTGCAGGCATGCCA	
⋆MtR4-28-854/CDH2(F)	ATGCGACCcGCAaatATGCCA	pTAL-R4-28-854/CDH2
⋆WtR1-15-286/IGSF11(F)	TTTGCTGCCTTTGTCCTGTAG	
⋆MtR1-15-286/IGSF11(F)	TTTGCTattTTTaTCCTGTAG	pTAL-R1-15-286/IGSF11
⋆HMT	AACGGACCGCCCGCGGCCCGT	

Note: ⋆ Only sense strands for oligonucleotides used in EMSA were shown; underlined parts were the putative binding sites, and mutated nucleotides were denoted as the lowercase letters. (F): forward primer; (R): reverse primer.

### Transient transfections and reporter gene assays

In 100 mm culture dishes, HBL-100 and MDA-MB-231 cells were grown to 80% confluence and transfected with 12 μg of pCMV-Myc-AP-2γ or pCMV-Myc, MDA-MB-453 cells were grown to 85% confluence and transfected with 600 pmol of AP-2γ siRNA or scramble siRNA. Twenty four hours after transfection, the cells were collected to prepare total RNA and protein lysates for quantitative real-time PCR and immunoblot analysis of reporter genes. The siRNAs (purchased from Shanghai GenePharma) were as follows: AP-2γ siRNA (sense), 5'-GCUCUACGUCUAAAU ACAATT-3'; AP-2γ siRNA (antisense), 5'-UUGUAUUUAGACGUAGAGCTG-3'; scramble siRNA (sense), 5'-UUCUCCGAACGUGUCACGUTT-3'; scramble siRNA (antisense), 5'-ACGUGACACGUUCGGAGAATT-3'.

For luciferase assays, MDA-MB-453, MDA-MB-231 and HBL-100 cells were seeded at an initial density of 2 × 10^5 ^cells/well in 12-well plates, then transfected with 600 ng of the luciferase reporter constructs and 600 ng of pCMV-Myc-AP-2γ or pCMV-Myc. 400 ng of pCMV-LacZ were used as internal reference to monitor transfection efficiency. Twenty four hours after transfection, the cells were lysed, and luciferase assays were performed using the Promega Luciferase Assay system. All data were from duplicate of at least three independent experiments.

### Electrophoretic gel mobility shift assays

The oligonucleotides containing wild-type or mutated binding sites of transcription factor AP-2γ were synthesized; and the wild-type consensus sequences of AP-2γ binding site on the promoter of human metallothionein IIa was used as competitor DNA (Table 1). The forward oligonucleotides were end-labeled with [γ-^32^P] ATP using T4 polynucleotide kinase, and combined with unlabeled reverse oligonucleotide after purification (QIAquick Nucleotide Removal Kit). The annealing mixture was heated to 95°C for 10 min, and cooled down slowly to room temperature to form probes. Vector pGEX-4T-2 (Amersham) was used to express GST-AP-2γ fusion proteins. 5 pmol of labeled probes were incubated with 50 pmol of purified recombinant GST-AP-2γ protein for 20 min at 37°C in 15 μl of reaction buffer: 10 mM HEPES (pH 8.0), 60 mM KCl, 4 mM MgCl2, 0.1 mM EDTA, 100 μg/ml BSA, 0.25 mM DTT, and 10% glycerol; then were resolved on 6% non-denaturation polyacrylamide gels. After drying, gels were exposed to Kodak X-ray film at -80°C, and signals were detected by autoradiography. Competition reactions were performed by pre-incubating the GST-AP-2γ protein with 10- or 50-fold of excess unlabeled competitor DNA prior to addition of the radioactive probes.

### Quantitative real-time PCR (Q-RT-PCR)

Total RNA was isolated from mammary-derived cell lines with Trizol reagent (Invitrogen, Carlsbad, CA), and contaminated chromosomal DNA was removed by RNase-free DNase treatment. cDNA was synthesized from 2 μg of total RNA using Oligo (dT)_18 _Primer according to the manufacturer's instruction (Fermentas, Vilnius, Lithuania). Then gene expressions were analyzed by quantitative real-time PCR (Q-RT-PCR) using 5 ng of reverse-transcribed total RNA template. The amplified products were detected with SYBR Green (ABI, San Francisco, USA) in combination with a melting curve analyses to ensure specificity (7900 HT Fast Real-time PCR System), and Delta Ct Method was used for quantification. The primer sequences were as follows: AP-2γ sense, 5'-GCCATCTCATTTCGTCCTCC-3'; AP-2γ antisense, 5'-GCCATCTCATTTCGTCCTCC-3'; ErbB2 sense, 5'-GAGACCCGCTGAACAATACC-3'; ErbB2 antisense, 5'-TGGATCAAGACCCCTCCTTT-3'; IGSF11 sense, 5'-GACTTGGGCCAATCTTTCTC-3'; IGSF11 antisense, 5'-TGTGGTGATGACCCTCTGTT-3'; CDH2 sense, 5'-ACCAGGTTTGGAATGGGACA-3'; CDH2 antisense, 5'-ACATGTTGGGTGAAGGGGTG-3'; HPSE sense, 5'-TTGCTATCCGACACCTTTGC-3'; HPSE antisense, 5'-CACGCTTGCCATTAACACCT-3'; β-actin sense, 5'-GCGCGGCTACAGCTTCA-3'; β-actin antisense, 5'-CTTAATGTCACGCACGAT TTCC-3'. The reactions yielded a 433-bp PCR product for AP-2γ, a 109-bp PCR product for ErbB2, a 119-bp PCR product for IGSF11, a 156-bp PCR product for CDH2, a 210-bp PCR product for HPSE and a 60-bp PCR product for β-actin. All experiments were preceded in triplicate.

### PCR amplification

Standard PCR was carried out to determine the specificity of AP-2γ binding DNA fragments derived from the AP-2γ-ChIP and normal rabbit IgG negative control. The ChIP-DNA fragments from MDA-MB-453 cells were amplified under conditions of 16.6 mM (NH4)_2_SO4, 0.67 mM Tris (pH8.8), 6.7 mM MgCl_2_, 10 mM β-mercaptoethanol, 10% dimethyl sulfoxide, 1.5 mM nucleotides, and 1.25 U of Taq polymerase. PCR products were resolved on a 1% agarose gel, and stained with ethidium bromide. The primer sequences were as follows: R4-28/CDH2-271 bp, (F) 5'-ATTTCCAAGTGGCTGAGGCA-3', (R) 5'-TGAACCATTGATTACGCCCA-3'; R1-15/IGSF11-291 bp, (F) 5'-GCATTCTGTCATTTTGGGTC-3', (R) 5'-CAACAACAATGGGTTCCTTC-3'; R3-36/HPSE- 260 bp, (F) 5'-GGCAGAAGGGCATAGTGTCT-3', (R) 5'-CAGGGATGTGGGCTAAGTGA-3'; R3-1/ErbB2-227 bp, (F) 5'-GTAAAA CGACGGCCAGT-3', (R) 5'-AGGAAACAGCTATGAC-3'.

### Western blot

Cell pellets were lysed in RIPA buffer (50 mM Tris-HCl (pH 7.2), 150 mM NaCl, 1% (w/v) Triton X-100, 1% (w/v) sodium deoxycholate, 0.1% (w/v) SDS) containing protease inhibitors (Boehringer cocktail). Then the proteins were subjected to electrophoresis on 10% SDS- polyacrylamide gel, and detected by western blot using rabbit polyclonal antibodies: anti-AP-2γ (sc-8977, 1:200), anti-ErbB-2 (sc-284, 1:200), anti-HPSE (sc-25825, 1:200), and anti-CDH2 (BA0673, 1:200). GAPDH (Mab-2005079, 1:2000) was used as loading control.

## Results

### ChIP assay identifies novel genomic binding sites for AP-2γ associated with genes involving carcinogenesis in MDA-MB-453 breast cancer cells

**Figure 1:**
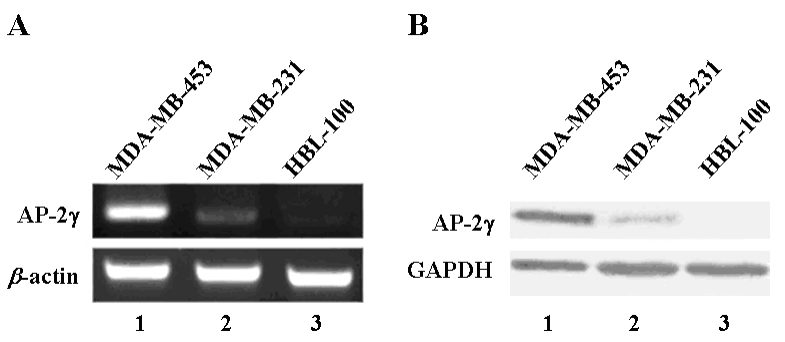
**AP-2γ highly expressed in MDA-MB-453 cells, but only lowly expressed in MDA-MB-231 and HBL-100 cells**. **A**. Expression of AP-2γ detected by RT-PCR. **B**. Expression of AP-2γ detected by immunoblot analysis. *Lane 1*, MDA-MB-453 cells. *Lane 2*, MDA-MB-231 cells. *Lane 3*, HBL-100 cells. Housekeeping genes β-actin and GAPDH were used as internal controls.

To investigate the expression of AP-2γ in breast cancer cell lines, we performed RT-PCR (Figure 1A) and immunoblot analysis (Figure 1B) in MDA-MB-453, MDA-MB-231 and HBL-100 cells. The results indicated that AP-2γ was highly expressed in MDA-MB-453 cells, but only lowly expressed in MDA-MB-231 and HBL-100 cells.

So we used ChIP assay to isolate AP-2γ binding sites in its target genes from MDA-MB-453 cells. Protein/DNA complexes were immunoprecipitated using polyclonal anti-AP-2γ antibody; Normal rabbit IgG and without antibody were taken as negative controls. The immunoprecipitated DNA fragments were cloned into pUC19 vector for blue/white screening. Forty clones from AP-2γ-ChIP-derived DNA fragments were obtained and sequenced, whereas no clones were found from negative controls.

The inserted AP-2γ-ChIP-derived DNA fragments ranged from 300 bp to 2 kb; their sequences were mapped to human genome using BLAST algorithm (Additional file 1). Genes near to these 40 ChIP fragments were determined using GeneHuggers, a bioinformatics program which enables precise selection of subsequence regions from records of the RefSeq human genome database based on diverse criteria. Through combination search of BLAST and GeneHuggers, we found multiple clones for eight fragments, and one of them was well known to contain AP-2γ binding sites before. 20 novel AP-2γ-ChIP fragments were mapped in proximity to known or predicted genes (Table 2). Within them, six fragments located at the intronic region of a gene, three fragments located at the downstream region of a gene, but about sixty percent (12 of 21) of fragments located at the upstream region of a gene; this suggested a binding preference of AP-2γ is the upstream region of a gene.

**Table 2:** Categories of putative AP-2γ target genes

Fragment/Gene Carcinogenesis	GeneBank accession no	Chromosome location	Distance from gene (kb)	location
R1-15/IGSF11	NT_005612.15	3q13.3	*	Intron 3
R3-36/HPSE	NT_016354.18	4q21	23	5'
R4-28/CDH2	NT_010966.13	18q11.2-12	103	5'
R3-1/ErbB2	NT_010755	17q11.2-q12	1	5'
Catalytic activity/metabolism				
R1-7/GALK1	NT_010641.15	17q24	4.5	5'
R4-21/MAT2B	NW_922784.1	5q34	1	5'
R1-11/UPP1	NT_007819.16	7p12-13	30	3'
R1-2/SQRDL	NT_010194.16	15q21	50	3'
R4-25/ACOT7	NT_021937.18	1p36.2	*	Intron 2
others				
R4-8/HIVEP1	NW_922984.1	6p23-24	42	5'
R3-2/CNTNAP2	NW_923707.1	7q35-36	*	Intron 1
R3-14/APOL3	NT_011520.11	22q12-13	16.5	5'
R2-13/AAK1	NT_022184.14	2p12-14	3	5'
R1-9/SEMA6D	NW_925884.1	15q12	1	5'
R3-26/HEL308	NW_922162.1	4q21	48	3'
R1-26/EMR1	DQ217942.1	19p13.2	1	5'
R3-35/ALG14	NW_921795.1	1p21-22	*	Intron 1
unknown				
R1-1/SIPA1L2	NT_004559.13	1q26	*	Intron3
R4-20/FRY	NT_024524.13	13q12-13	*	Intron 3
R2-2/TMEM148	NT_010498.15	16q22	49	5'
R2-7/LOC401864	NT_010498.15	16q24	1	5'

* The fragment is part of the gene.

The genes near to AP-2γ-ChIP fragments were considered as potential targets for AP-2γ binding and regulation. They were grouped into different functional categories (Table 2), which involved in the regulation of carcinogenesis, metabolism or others. Fragments appeared in multiple clones were R1-7/GALK1, R3-36/HPSE, R4-8/HIVEP1, R3-14/APOL3, R2-2/TMEM148, R2-7/LOC401864, R3-1/ErbB2 and R4-28/CDH2, in which R3-1/ErbB2 was well known to contain AP-2γ binding sites before [1,25]. Genes involved in the regulation of carcinogenesis include IGSF11, HPSE, CDH2 and ErbB2 (Additional files 2, 3, 4 and 5). Considering the role of AP-2γ in carcinogenesis, we chose these four genes for further investigation to elucidate their functions in breast cancer.

### AP-2γ upregulates the transcription activity through binding to ErbB2, CDH2, HPSE and IGSF11

**Figure 2:**
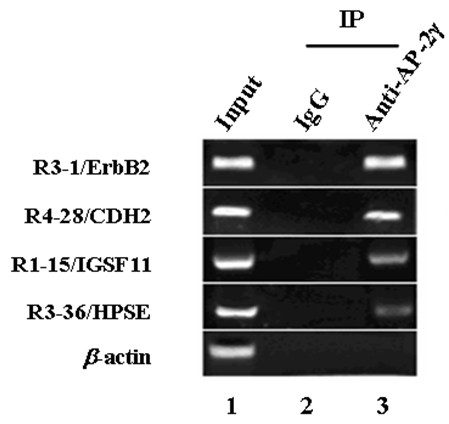
**AP-2γ binds to genomic DNA fragments near to ErbB2, CDH2, HPSE and IGSF11 genes in vivo**. Normal rabbit IgG and rabbit polyclonal anti-AP-2γ antibody were used to immunoprecipitate protein-bound DNA in MDA-MB-453 cells. The immunoprecipitated genomic DNA was subjected to PCR amplification using primers flanking the AP-2γ response elements. Genomic DNA (Input) without immunoprecipitation was used as positive controls, and housekeeping gene β-actin was used as negative control.

At least two independent ChIP experiments were performed to confirm the binding of AP-2γ to the four isolated ChIP fragments near to genes ErbB2, CDH2, HPSE and IGSF11, the related PCR products were detected from input samples and AP-2γ-ChIP-derived DNA samples, but not from IgG-ChIP-derived DNA samples (Figure 2).

**Figure 3:**
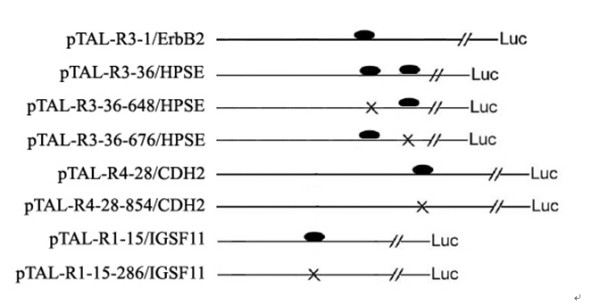
**Diagram of luciferase vectors of wild-type and mutant AP-2γ-ChIP-fragments**. Wild-type or mutant AP-2γ-ChIP-fragment was cloned into the pTAL-Luc vector for luciferase assay. The wild-type binding sites of AP-2γ were indicated by oval, and the mutated sites were indicated by cross (X).

Transcription factor AP-2γ binding sites near to genes ErbB2, CDH2, HPSE and IGSF11 were predicted by JASPAR program and MatInspector program http://www.genomatix.de utilizing TRANSFAC matrices (core similarity 1.0 and matrix similarity 0.9). We found one consensus binding site in R3-1/ErbB2 (644 to 652), two consensus binding sites in R3-36/HPSE (648 to 656, and 676 to 684), one consensus binding site in R4-28/CDH2 (854 to 862), and one consensus binding site in R1-15/IGSF11 (286 to 294). To clarify how AP-2γ regulates the gene expression of ErbB2, CDH2, HPSE and IGSF11 through binding to the predicted sites, wild-type and mutant of AP-2γ-ChIP fragment luciferase vectors were generated through appropriate restriction enzyme digestion and overlapping extension PCR (Figure 3). Because ErbB2 has previously been characterized as an AP-2γ target gene [21], the mutant of ErbB2 luciferase vector was not included.

**Figure 4:**
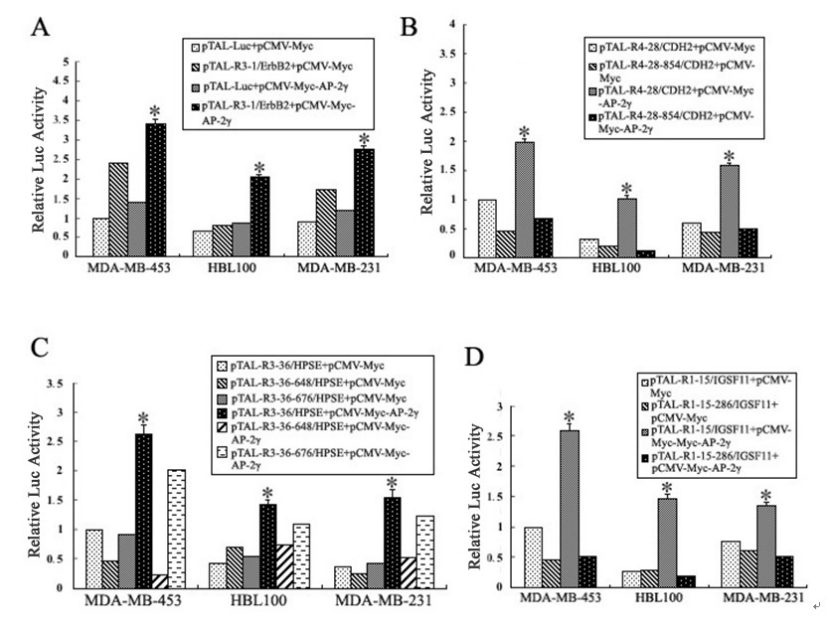
**Over-expression of AP-2γ significantly upregulated the transcription activities of ErbB2, CDH2, HPSE and IGSF11**. Luciferase vectors of wild-type or mutant AP-2γ-ChIP-fragments were cotransfected with pCMV-Myc-AP-2γ or pCMV-Myc into MDA-MB-453, MDA-MB-231, and HBL-100 cells, and luciferase gene expression was assayed. **A**. Wild-type R3-1/ErbB2 was upregulated by AP-2γ in different breast cancer cells. **B**. Mutation on AP-2γ binding site of CDH2 abrogated the upregulation by AP-2γ. **C**. Disruption of binding site on R3-36/HPSE (648–656) reduced the upregulation of HPSE by AP-2γ; "however the binding site on R3-36/HPSE (676–684) is not functional. **D**. Mutation of binding site R1-15-286/IGSF11 inhibited the upregulation of IGSF11 by AP-2γ in different breast cancer cells. The relative luciferase activity in each sample was normalized and presented as mean ± SD from triplicated independent experiments. (* means P < 0.01).

Reporter gene assays were performed to check the transcription activity. Related wild-type or mutant luciferase constructs were cotransfected with pCMV-Myc-AP-2γ or pCMV-Myc into MDA-MB-453, MDA-MB-231, and HBL-100 cells; and the reporter gene luciferase activity was assayed. pCMV-LacZ was also cotransfected in all experiments, and β-galactosidase activity was used to normalize transfection efficiency. As shown in Figure 4, AP-2γ upregulated the expression of four genes (R3-1/ErbB2, R4-28/CDH2, R3-36/HPSE and R1-15/IGSF11) to about 2~3 folds (Bars marked by asterisk). However, the binding site of R3-36/HPSE (676 to 684) is not functional; the decrease of luciferase activity is not statistically significant (Figure 4C).

### Identification of putative AP-2γ binding sites on genes CDH2, HPSE and IGSF11

**Figure 5:**
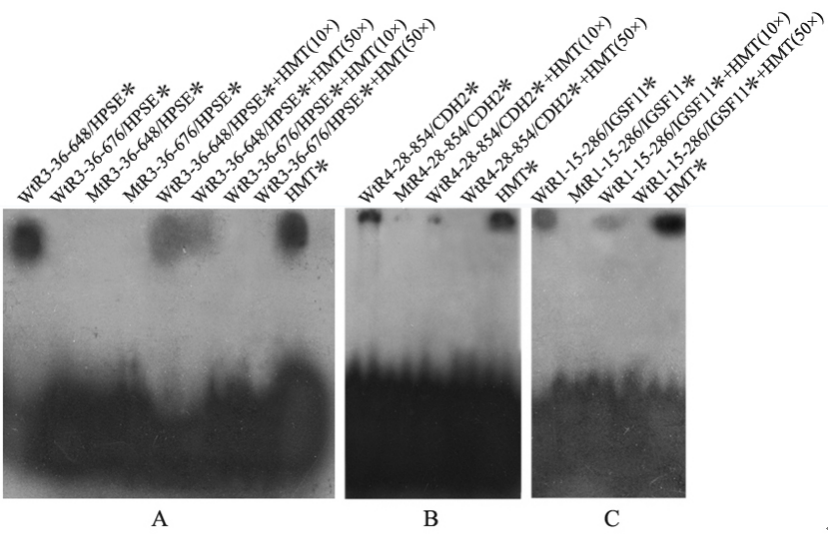
**Electrophoretic gel mobility shift assays**. GST-AP-2γ fusion protein was incubated with ^32^P-labeled wild-type or mutated oligonucleotides (marked by asterisk). The protein-DNA bindings were also confirmed by competition experiments with unlabeled AP-2γ consensus binding sequence in human metallothionein IIa promoter (HMT). The retarded bands showed positive binding results, and the intense bands at the bottom were unbound labeled probes. Data confirmed the binding elements on HPSE, CDH2 and IGSF11.

To further define the sequence elements for AP-2γ binding, we conducted electrophoretic gel mobility shift assays (EMSA) using ^32^P-labeled probes corresponding to the potential AP-2γ binding motifs in AP-2γ-ChIP fragments. As shown in Figure 5, purified recombinant GST-AP-2γ protein binds to wild-type oligonucleotides WtR3-36-648/HPSE, WtR4-28-854/CDH2 and WtR1-15-286/IGSF11, but not to mutated oligonuleotides MtR3-36-648/HPSE, MtR4-28-854/CDH2 and MtR1-15-286/IGSF11. The specificity of such bindings was also confirmed by competition experiments with excess wild-type consensus sequence of AP-2γ binding site in HMTIIa promoter (HMT). After treatment with 10- or 50-fold of excess unlabeled oligonucleotides HMT, these DNA-protein bindings were markedly decreased. Both wild-type oligonucleotides WtR3-36-676/HPSE and mutated oligonuleotides MtR3-36-676/HPSE did not produce retarded bands, indicating that the sequence element HPSE (676–684) is not the functional binding site of AP-2γ. Because the mutation of the AP-2γ binding site in ErbB2 has also previously been demonstrated to lead to a significant decrease of their binding in an EMSA experiment [26], ErbB2 was not included in **5**

### AP-2γ-dependent regulation of candidate target genes at mRNA and protein level

**Figure 6:**
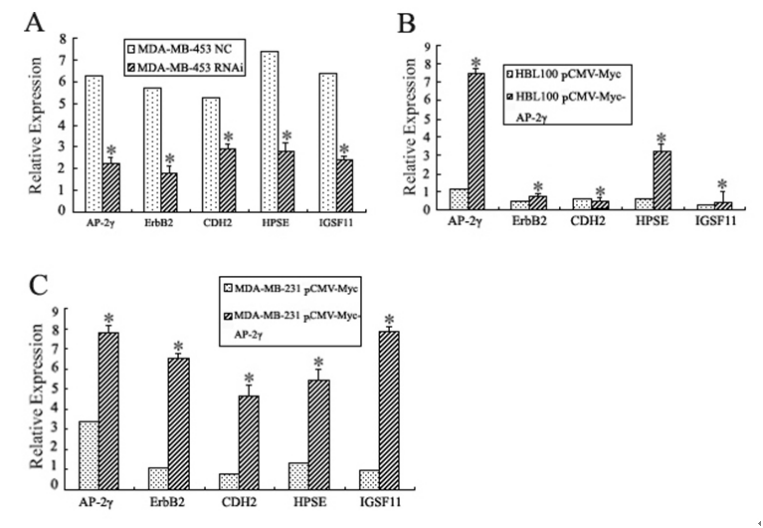
**AP-2γ affected the expressions of ErbB2, CDH2, IGSF11 and HPSE at mRNA levels**. The mRNA levels of ErbB2, CDH2, IGSF11 and HPSE were examined using Q-RT-PCR method while suppressing endogenous AP-2γ expression in MDA-MB-453 cells (**A**); or overexpressing AP-2γ in HBL-100 cells (**B**) and in MDA-MB-231 cells (**C**). β-actin was used as an internal control to normalize the expression of each target mRNA. Relative gene expression was presented as mean ± SD from triplicated independent experiments. (* means *P *< 0.01)

To make sure whether the genes associated with the fragments identified by ChIP assays are functional targets of AP-2γ or not, endogenous AP-2γ suppression experiment by RNA interference was performed in MDA-MB-453 cells and overexpression experiment by transient transfection of AP-2γ were performed in HBL-100 cells and MDA-MB-231 cells (Figure 6). Q-RT-PCR data from at least three independent experiments were calculated with Delta Ct Method.

In MDA-MB-453 cells transfected with AP-2γ siRNA, AP-2γ mRNA levels showed an about 3-fold decrease (Figure 6A). Accordingly, the mRNA levels of ErbB2, CDH2, HPSE and IGSF11 also dramatically decreased 2~3-fold. In HBL-100 cells, while AP-2γ mRNA levels increased about 7.5-fold (Figure 6B), the HPSE mRNA level increased, but the ErbB2, CDH2 and IGSF11 mRNA levels remained unchanged. In MDA-MB-231 cells, while AP-2γ mRNA levels increased about 2.5-fold (Figure 6C), the mRNA levels of ErbB2, CDH2, HPSE and IGSF11 were then dramatically up-regulated about 4~8-fold.

**Figure 7:**
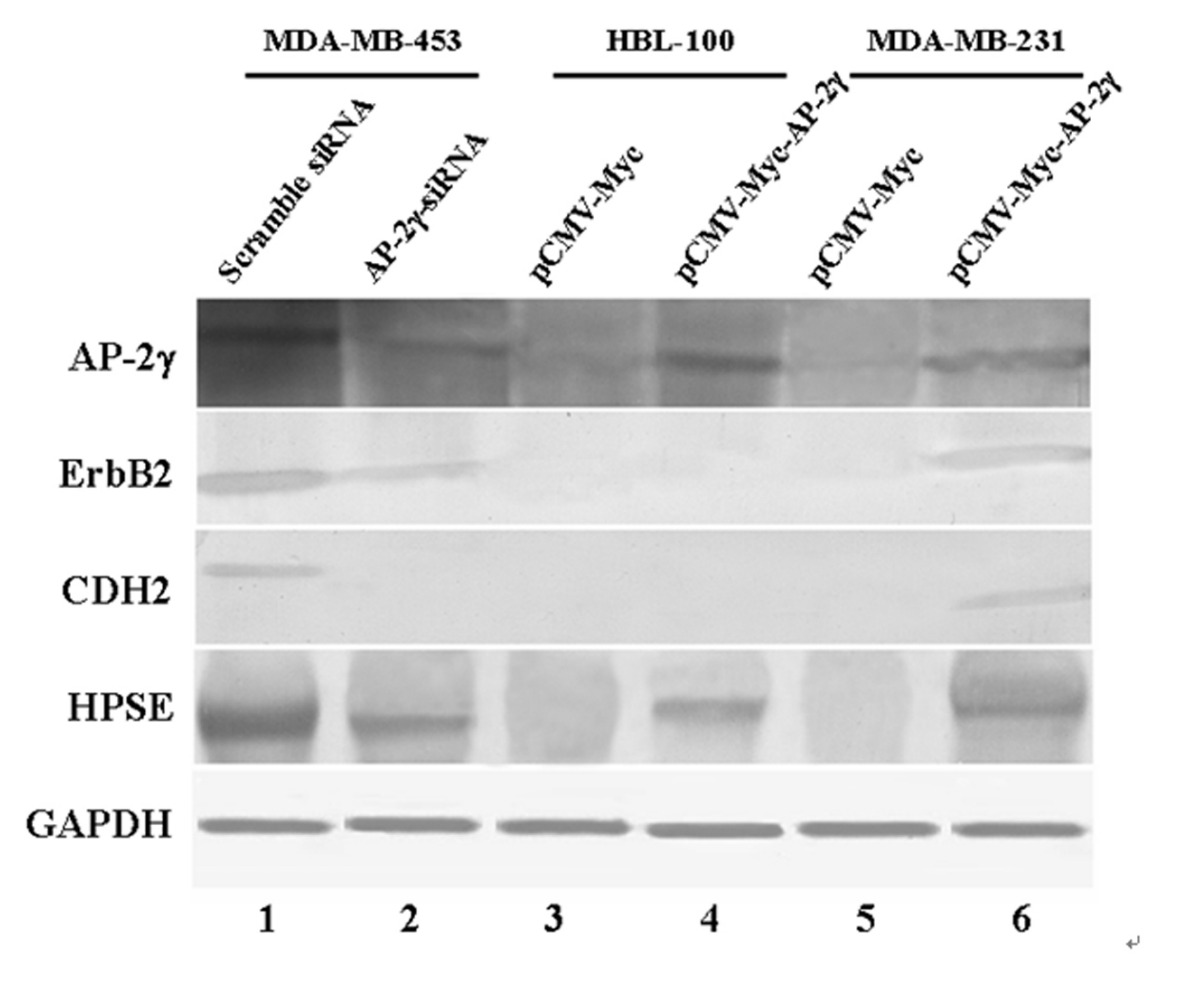
**AP-2γ expression alterations changed the ErbB2, CDH2, and HPSE protein level in breast cancer cells**. MDA-MB-453 cells were transfected with negative control siRNA(*Lane 1*) and with AP-2γ siRNA (*Lane 2*). HBL-100 cells were transfected with negative control pCMV-Myc (*Lane 3*) and with pCMV-Myc-AP-2γ (*Lane 4*). MDA-MB-231 cells were transfected with negative control pCMV-Myc (*Lane 5*) and with pCMV-Myc-AP-2γ (*Lane 6*). The housekeeping gene GAPDH was used as an internal control.

In MDA-MB-453 cells transfected with AP-2γ siRNA, following with the decrease of AP-2γ protein level, the protein levels of ErbB2, CDH2 and HPSE were also dramatically down-regulated (Figure 7, *Lane 2*). While overexpressing AP-2γ protein in MDA-MB-231 cells, the protein levels of ErbB2, CDH2 and HPSE were dramatically increased (Figure 7, *Lane 6*). However, in HBL-100 cells, overexpression of AP-2γ protein only significantly increased HPSE protein level, the protein level of ErbB2 and CDH2 remained unchanged (Figure 7, *Lane 4*). As IGSF11 antibody is not commercially available, we didn't check the protein level changes by the alteration of AP-2γ expression.

## Discussion

In post-genome era, studying the gene expression and regulation in eukaryotic cells becomes more and more important. The genomic DNA exists in eukaryotic cells as chromatin, which is a dynamic structure could control the access of transcription factors to DNA binding sites, influences the interactions between different transcription factors, and mediates their subnuclear localization [27,28]. So studying the interaction between proteins such as transcription factors and genomic DNA at its chromatin status is very critical for elucidating the regulation mechanism of gene expression. Chromatin immunoprecipitation (ChIP) is a method used to determine the location of DNA binding sites on the genome for a particular protein of interest. This technique gives a picture of the protein-DNA interactions that occur inside the nucleus of living cells or tissues. Although there are more methods to study the interactions between transcription factors and genomic DNA, this is the only real *in vivo *approach. Using ChIP assay to isolate AP-2γ binding sites from accessible regions of native chromatin in MDA-MB-453 breast cancer cells allowed us to identify a number of AP-2γ target genes as well as to find out how AP-2γ affect these gene expressions in living cells.

In this study, we have isolated forty clones from AP-2γ-ChIP-derived genomic DNA fragments; we did not obtain any clones from the negative controls indicating a low rate of false positive clones. Among the forty clones, eight fragments were found within multiple clones, and one of these fragments (R3-1/ErbB2) has previously been shown to contain functional AP-2γ binding sites. The binding of AP-2γ to the isolated ChIP fragments was confirmed using independent ChIP assays (Figure 2), emphasizing the reliability of our findings. The four AP-2γ binding fragments examined in more detail (associated with genes ErbB2, CDH2 IGSF11 and HPSE) could all be stimulated by co-expression of AP-2γ in reporter assays (Figure 4), and were able to bind to recombinant protein GST-AP-2γ (Figure 5).

All four of the genes validated here were found to be positively regulated by AP-2γ. However, examination of the regulation of expression of the remaining 17 novel candidate genes, using Q-RT-PCR to examine both AP-2γ over-expressing MDA-MB-231 cells and AP-2γ silenced MDA-MB-453 cells, has shown that some of the genes (LOC401864, SEMA6D, STPA1L2, and FRY) identified in the screen can be negatively regulated by AP-2γ (Additional file 6). Thus, the ChIP-cloning assay described here has successfully identified both activated and repressed genes; however more work is still required to further define how AP-2γ regulates these 17 potential target genes.

AP-2γ has been reported to participate into the regulation of breast cancer initiation and progression [21,29,30]. And it has also been suggested to be a marker of testicular and germ cell malignancies [19,31-33]. These findings show the biological importance of AP-2γ in human neoplasia. In our study, the AP-2γ-ChIP fragments were obtained from human breast cancer cell line MDA-MB-453 with high malignant degree; knock-down of AP-2γ expression in MDA-MB-453 cells by siRNA decreased the expressions of ErbB2, CDH2, IGSF11 and HPSE (Figures 6A and 7), but overexpression of AP-2γ in MDA-MB-231 cells elevated their mRNA and protein levels (Figures 6C and 7). They are the direct regulatory targets of AP-2γ. Current studies suggested that these four genes are all involved in carcinogenesis. In human breast cancer, ErbB-2 is overexpressed in 25–30% of all cases, and is representing a clinical marker of poor prognosis [34]; CDH2 promotes tumor cell survival, migration and invasion, and its high expression is often associated with poor prognosis [35]; IGSF11 expression is elevated in colorectal cancers, hepatocellular carcinomas and intestinal-type gastric cancers [36]; HPSE plays a key role in the metastasis of solid tumors as well as mediating tumor angiogenesis by both directly promoting endothelial cell migration and indirectly releasing proangiogenic molecules from the ECM [37-39]. They probably mediate the effects of AP-2γ on the tumor progression. In human mammary carcinoma, AP-2γ may also functionally interact with these genes to influence tumor progression besides transcriptionally activating their expressions.

Overexpression of AP-2γ in HBL-100 and MDA-MB-231 presented different results on regulating ErbB2, CDH2 and IGSF11 (Figures 6B and 6C), indicating that the transcriptional regulation network are complex. Similarly, although the regulation patterns for most of the other 17 identified genes were revealed identical using the two complementary approaches – overexpressing AP-2γ in MDA-MB-231 and silencing AP-2γ in MDA-MB-453 (Additional file 6), upon closer examination, there are some inconsistencies. In particular, for AAK1 (Adaptor protein 2-associated kinase 1), overexpression of AP-2γ results in a 4-fold increase in AAK1 expression, while there is no decrease in AAK1 expression in the siRNA experiment. This also implies the complexity of the regulation of AAK1 by AP-2γ. Transcription factors (TFs) directly control gene expressions by interacting with cis-elements, but transcription regulators (TRs) only assist to control gene expression through interacting with TFs, remodeling chromatin, or other mechanisms. Both types of proteins constitute master controllers of dynamic transcriptional networks. In different cell lines, transcriptional regulation networks might be different.

## Conclusion

In summary, AP-2γ coordinates the expressions of a network of genes, involving in carcinogenesis, especially in breast cancers. Our data might contribute to better understanding the controversial findings concerning the role of transcription factor AP-2γ in human breast cancer. The novel AP-2γ target genes could serve as therapeutic targets for anticancer therapy.

## Competing interests

The authors declare that they have no competing interests.

## Authors' contributions

AH, XX and DR contributed equally to this work. Correspondence should be addressed to Prof. Jian Zhang. All authors have read and approved the final manuscript.

## Pre-publication history

The pre-publication history for this paper can be accessed here:

http://www.biomedcentral.com/1471-2407/9/279/prepub

## Supplementary Material

Additional file 1 The relative positions of the fragments (R1-15/IGSF11, R3-36/HPSE, R4-28/CDH2, and R3-1/ErbB2) obtained from the ChIP-cloning assay. Click here for file

Additional file 2 The sequence of R1-15/IGSF11 fragment. Click here for file

Additional file 3 The sequence of R3-36/HPSE fragment. Click here for file

Additional file 4 The sequence of R4-28/CDH2 fragment. Click here for file

Additional file 5 The sequence of R3-1/ErbB2 fragment. Click here for file

Additional file 6 **The examination of expression regulation of other 17 identified novel genes by AP-2γ using Q-RT-PCR**. **A**. Overexpressing AP-2γ by transient transfection in MDA-MB-231 cells. **B**. Silencing AP-2γ by RNA interference in MDA-MB-453 cells. Click here for file
