# Supplementary figures and images for: Identification of target genes of transcription factor activator protein 2 gamma in breast cancer cells
Source: BMC Cancer. 2009 Aug 11;9:279. doi: 10.1186/1471-2407-9-279 (PMC3224728; doi:10.1186/1471-2407-9-279)

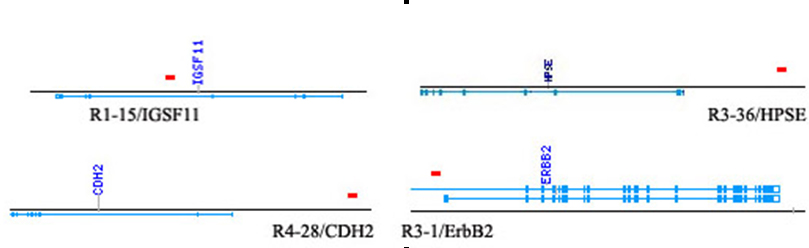

Supplement: Additional file 1 — The relative positions of the fragments (R1-15/IGSF11, R3-36/HPSE, R4-28/CDH2, and R3-1/ErbB2) obtained from the ChIP-cloning assay. [file 1471-2407-9-279-S1.jpeg]

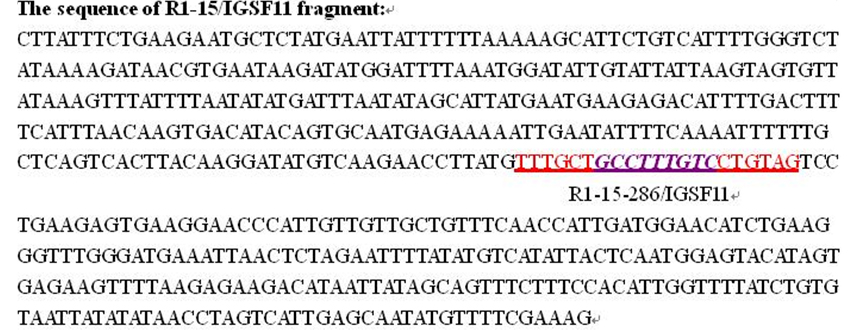

Supplement: Additional file 2 — The sequence of R1-15/IGSF11 fragment. [file 1471-2407-9-279-S2.jpeg]

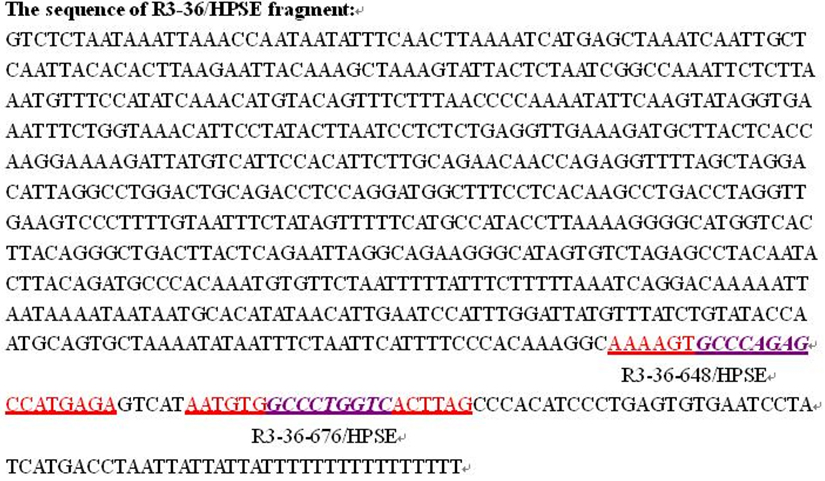

Supplement: Additional file 3 — The sequence of R3-36/HPSE fragment. [file 1471-2407-9-279-S3.jpeg]

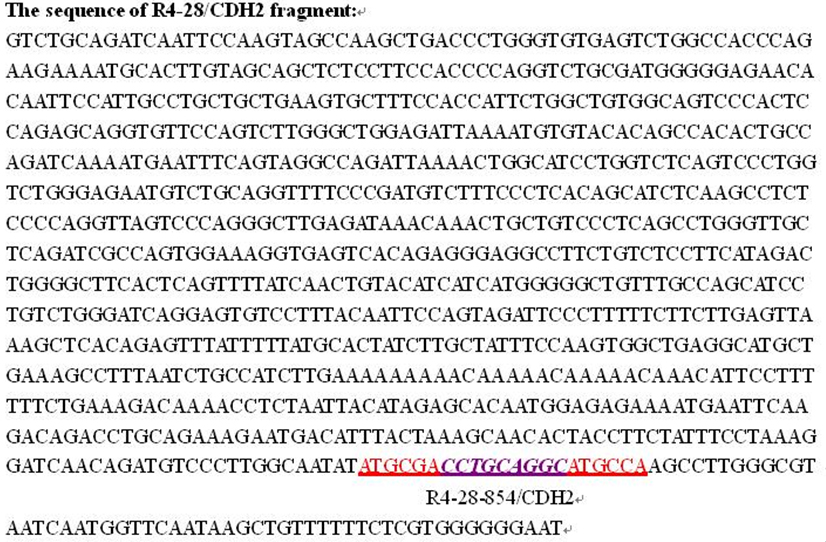

Supplement: Additional file 4 — The sequence of R4-28/CDH2 fragment. [file 1471-2407-9-279-S4.jpeg]

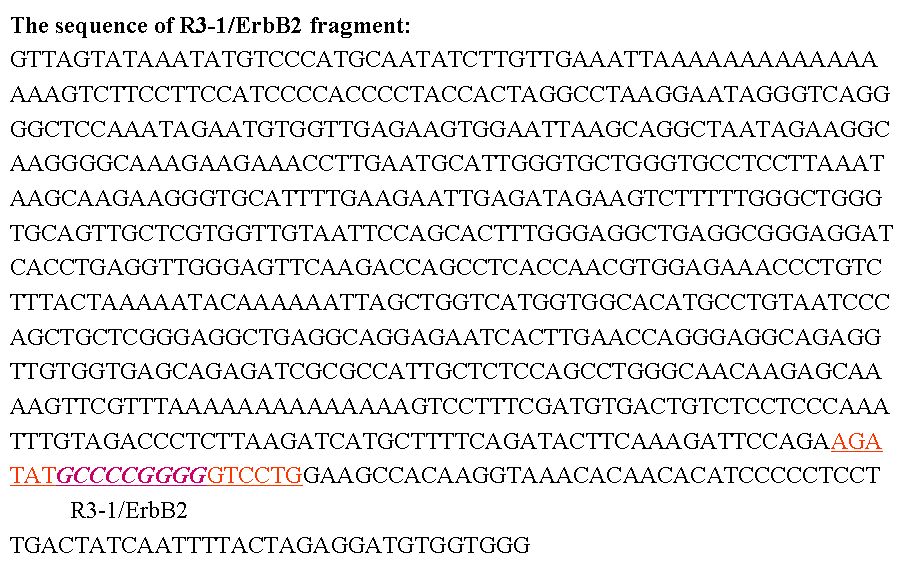

Supplement: Additional file 5 — The sequence of R3-1/ErbB2 fragment. [file 1471-2407-9-279-S5.jpeg]

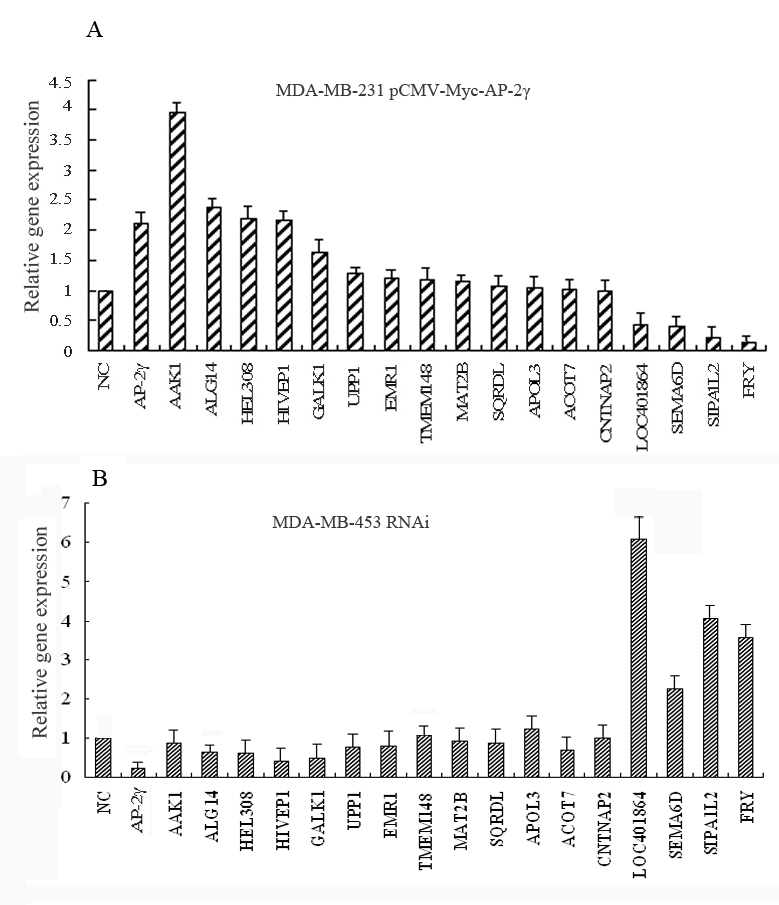

Supplement: Additional file 6 — The examination of expression regulation of other 17 identified novel genes by AP-2γ using Q-RT-PCR. A. Overexpressing AP-2γ by transient transfection in MDA-MB-231 cells. B. Silencing AP-2γ by RNA interference in MDA-MB-453 cells. [file 1471-2407-9-279-S6.jpeg]
